# Epigenetic control of mesenchymal stem cells orchestrates bone regeneration

**DOI:** 10.3389/fendo.2023.1126787

**Published:** 2023-03-06

**Authors:** Xiaofeng Wang, Fanyuan Yu, Ling Ye

**Affiliations:** ^1^ State Key Laboratory of Oral Diseases and National Clinical Research Center for Oral Diseases, West China Hospital of Stomatology, Sichuan University, Chengdu, China; ^2^ Department of Endodontics, West China Hospital of Stomatology, Sichuan University, Chengdu, China

**Keywords:** bone, bone regeneration, epigenetics, mesenchymal stem cells, stem cell

## Abstract

Recent studies have revealed the vital role of MSCs in bone regeneration. In both self-healing bone regeneration processes and biomaterial-induced healing of bone defects beyond the critical size, MSCs show several functions, including osteogenic differentiation and thus providing seed cells. However, adverse factors such as drug intake and body senescence can significantly affect the functions of MSCs in bone regeneration. Currently, several modalities have been developed to regulate MSCs’ phenotype and promote the bone regeneration process. Epigenetic regulation has received much attention because of its heritable nature. Indeed, epigenetic regulation of MSCs is involved in the pathogenesis of a variety of disorders of bone metabolism. Moreover, studies using epigenetic regulation to treat diseases are also being reported. At the same time, the effects of epigenetic regulation on MSCs are yet to be fully understood. This review focuses on recent advances in the effects of epigenetic regulation on osteogenic differentiation, proliferation, and cellular senescence in MSCs. We intend to illustrate how epigenetic regulation of MSCs orchestrates the process of bone regeneration.

## 1 Introduction

Although some bone fractures and defects (e.g., trauma, inflammation, tumors, etc.) can heal independently, those beyond the critical size cannot (1). Hence, various studies focus on accelerating bone regeneration. In these basic research and clinical trials, the involvement of mesenchymal stem cells (MSCs) has received much attention because both bone defect healing and biomaterials implanted in tissue engineering require the involvement of MSCs (2, 3). Although MSCs’ fate determination, cell proliferation, and cellular senescence are of great importance in bone regeneration (4, 5), the possible ways to modulate the biological behavior of MSCs to facilitate bone regeneration are yet to be fully understood.

Moreover, although some studies used modified culture methods and stimulation by exogenous signals to improve MSCs performance in bone regeneration (6, 7), these alterations are not genetically transmissible. They have a limited impact on bone regeneration research. Recently, epigenetic regulation of MSCs has received widespread attention because it can lead to heritable changes. The term “epigenetics” refers to the alteration of the phenotype of an organism by regulating the expression of genes without altering genetic material (DNA), and this alteration can be inherited. In other words, epigenetic regulation allows for stable changes in MSCs and can spread this effect through cell proliferation. Epigenetic control includes DNA methylation, histone modifications, non-coding RNA regulation, chromosomal spatial changes, etc. Given the epigenetic heritability, epigenetic regulation of stem cells can be transmitted horizontally and vertically during proliferation or differentiation, thus amplifying the signal.

**Figure 1:**
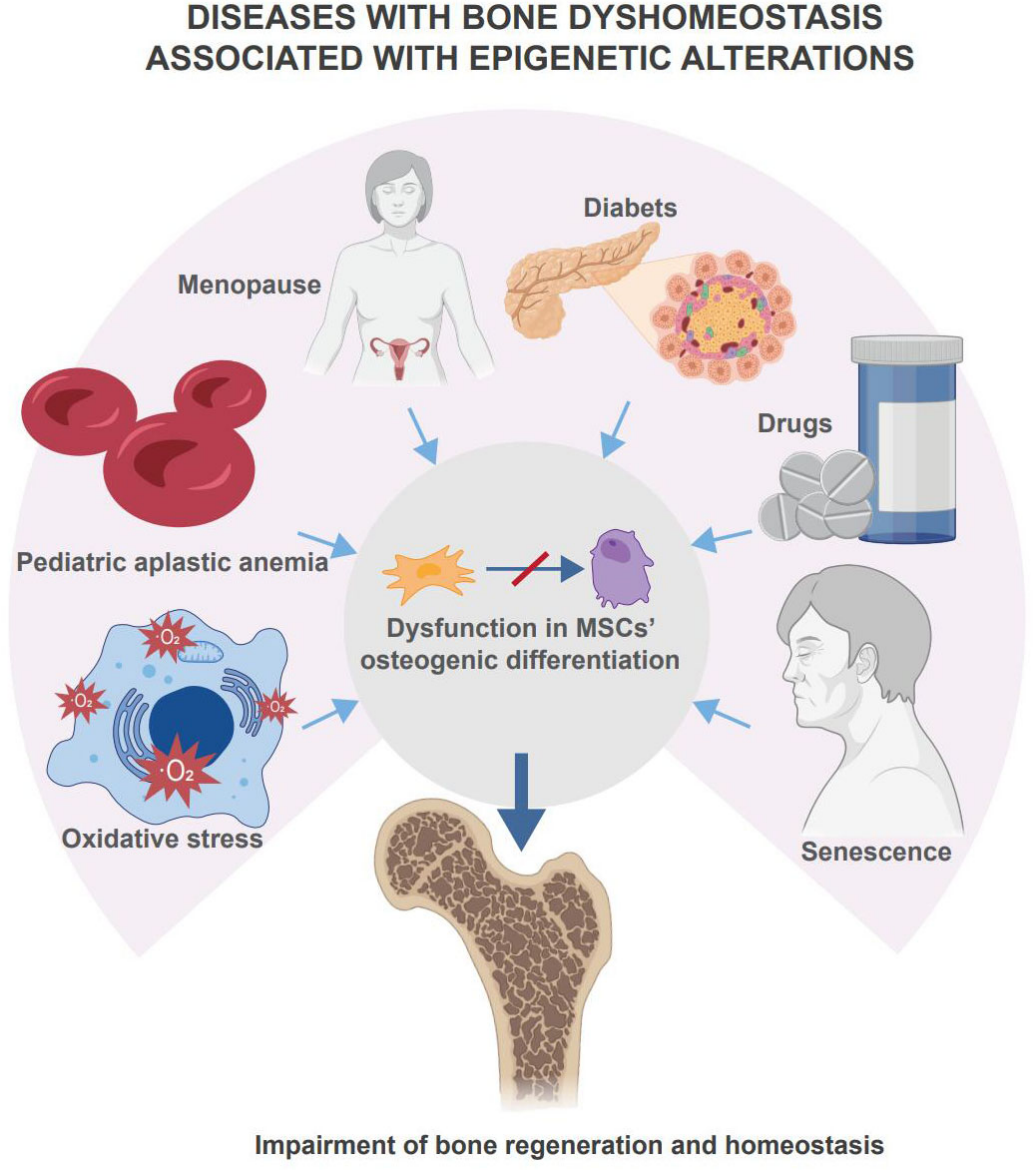
Disease with bone dyshomeostasis associated with epigenetic alterations. Multiple diseases, drugs, or body senescence will lead the skeletal system to bone dyshomeostasis. Among these situations, changes in epigenetic regulation affect the osteogenic differentiation of MSCs and mediate the loss of bone homeostasis and impairment of bone regeneration.

The role of epigenetic regulation in skeletal system development and homeostatic maintenance has been proven (8). Epigenetic disorders in MSCs are found in human bone homeostasis diseases such as osteoporosis due to diabetes, aging, etc. (9). Diseases with bone dyshomeostasis associated with epigenetic alterations are summarized in **Figure 1**. Mouse knockout models trigger disease models with abnormal bone homeostasis (10, 11). In these studies, epigenetic regulation of bone regeneration was achieved through the regulation of osteogenic differentiation, proliferative capacity, and cellular senescence in MSCs. This paper focuses on DNA methylation and histone modifications as representatives of the epigenetic regulation of MSCs in bone regeneration. Since related reviews provide detailed descriptions of miRNA regulatory roles and other types of epigenetic regulation, these will not be discussed in this paper.

## 2 MSCs and their role in bone regeneration

Mesenchymal stem cells (MSCs) are a population of cells that are derived from mesenchymal tissue and have the capacity for self-renewal and multidirectional differentiation. Obtaining sufficient numbers of MSCs for research requires *in vitro* isolation and culture, and in 2006, The International Society for Cellular Therapy proposed minimal criteria for MSCs. Firstly, the MSCs must maintain plastic adhesion under standard culture conditions. Second, MSCs must express CD105, CD73 and CD90 and lack the expression of CD45, CD34, CD14 or CD11b, CD79α or CD19 and HLA-DR surface molecules. Thirdly, MSC must differentiate *in vitro* into osteoblasts, adipocytes and chondrogenic cells (12). However, MSCs from different tissue sources exhibit surface marker heterogeneity, and even if derived from the same tissue, different isolation methods and culture media composition can have an impact on the properties of MSCs, thus subdividing them into different subgroups. Until now, no specific single marker has been accurately applied for the isolation and identification of MSCs. There is a growing recognition of the heterogeneity of the MSCs population. As a result, different MSCs may exhibit different osteogenic capacities *in vitro* and *in vivo*, a vague concept that greatly impairs the clinical translation of MSCs.

To address these issues, Paolo Bianco introduced the term ‘skeletal stem cells’(SSCs) to denote skeletal tissue-resident, self-regenerating and multipotent cells that give rise to cartilage, bone, haematopoietic support matrix and bone marrow adipocytes (13). The presence of SSCs has practical applications for the maintenance of bone homeostasis and the repair of bone defects. The identification of SSCs has more stringent conditions than MSCs, and it has been suggested that in addition to the validation of CFU and three-way differentiation capacity, self-renewal and multipotential properties should be tested by rigorous *in vivo* assays. Serial transplantation studies are the gold standard for validating true SSCs. Cells should be sorted with putative stem cell surface markers and xenografted, then assessed for bone and bone marrow component formation. Presumptive SSCs can be again isolated from the newly formed bone and then secondarily transplanted to test the ability to form intact skeletal components. Similarly, *in situ* analysis of the multipotential of putative SSCs by co-labelling with different spectral markers (14, 15). It is possible to represent SSCs and MSCs as distinct cell populations, and to some extent, SSCs can be considered as a more homogeneous subpopulation of MSCs (16). The use of “SSCs” is limited to self-regenerating and pluripotent cells of skeletal system origin and displaying clear surface markers. Despite the significance of SSCs, given their specific identification criteria, in the following, we have mainly used MSCs to refer to MSCs of various tissue origins.

MSCs with normal functions are essential for bone homeostasis and regeneration (17). For instance, MSCs isolated from patients with bone homeostasis disorders such as osteoporosis have impaired osteogenic and immunomodulatory capacities (9, 18–20). At the same time, functional MSCs impairment induces disease models and affects bone regeneration (21, 22). Therapies that target MSCs impairment can promote bone regeneration, and ways of using MSCs in biomaterials to accelerate bone healing continue to be devised. Although the involvement of MSCs is complex and not fully understood, it strongly indicates that MSCs have an essential role in bone regeneration.

During bone regeneration, local signals activate MSCs from their quiescent state. Peripheral MSCs hardly function in the bone regeneration process because most of this process relies on local stem cells, and the local signals that drive bone regeneration. Through cell proliferation, MSCs provide sufficient cell numbers; through intra- and extracellular signals, some daughter cells differentiate and get through fate determinations, which are the key osteogenetic functions of MSCs. Furthermore, the process is closely linked to cellular senescence, resulting in the permanent impairment of proliferative and differentiation capacities.

Bone regeneration is a dynamic process where new bone formation and remodeling interact and create a mutual balance of bone resorption and formation (23). This process can be observed in bone healing in adults (24). Closely related to bone regeneration, the bone defect healing process happens in two ways: primary healing and secondary healing (25). Secondary healing is more common, more similar to bone regeneration, and involves three phases: the inflammatory phase, the repair phase, and the reconstruction phase, with no clear division between these phases (17). After the injury, the hematoma forms rapidly, and inflammatory cells such as neutrophils and macrophages migrate to the injury site. Pro-inflammatory factors are highly expressed to achieve the anti-infection and phagocytosis of fracture debris (26). The inflammatory reaction peaks between 24 and 48 hours after an injury. Subsequently, the local environment gradually shifts from pro-inflammatory to pro-repair, and various cytokines associated with tissue repair begin to be consistently expressed. The hematoma is gradually replaced by mechanized tissue due to vascular and fibroblast ingrowth. Cytokines from platelets and macrophages provide recruitment signals for MSCs and promote their proliferation and osteodifferentiation. This process begins between 7 and 10 days after an injury. And MSCs undergo chondrogenic differentiation. The cartilage scab formed by chondrocytes highlights the bone healing and repair phases. Subsequently, osteoblast-associated cells derived from MSCs contribute to the substitution of hard scabs for cartilage scab, and cartilage scab becomes a biological scaffold for new bone mineralization and remodeling. However, with the cooperation of osteoclasts and osteoblasts, the Haversian system is completed after the reconstruction phase, and the bone forms a functionally matched structure.

## 3 Epigenetic control of MSCs in bone regeneration

The term “epigenetic regulation” refers to several processes that produce changes in heritable expressions without altering genomes. These processes include DNA methylation, histone modifications, non-coding RNA regulation, etc. Epigenetic regulation plays a regulatory role in several biological MSCs behaviors and is essential in maintaining homeostasis, cell fate determination, cell senescence, cell proliferation, and cell death in MSCs (9, 27–31). Considering several reviews on MSCs regulation by miRNAs, we focus on the effects of DNA methylation and histone modifications on MSCs proliferation, differentiation, and senescence in bone regeneration. Notably, multiple epigenetic regulations include crosstalk, but for ease of understanding, we will discuss the different types of regulation separately.

### 3.1 Epigenetic response of MSCs to extrinsic signals

Epigenetic enzymes are regulated by extrinsic microenvironmental, and cell transduction of these signals contributes to the MSCs’ response to external changes. Compared to 2D culture, 3D spheroid culture can significantly alter the cellular microenvironment, and this microenvironmental change can have direct effects on cells, such as changes in morphology, adhesion molecules, changes in intercellular contacts, and enhanced cell-cell and cell-matrix interactions induce significant modifications in cytoskeletal network within each cell of 3D aggregate. Changes in this microenvironment also affect epigenetics. For example, MSCs cultured in 3D spheroid for 2-3 days have an enhanced ability for clone formation and differentiation of neurons compared to 2D cultures. The expression levels of miRNAs involved in stem cell potency are altered and the levels of histone H3 acetylation in OCT4, SOX2 and NANOG promoter region K9 are elevated. Thus, 3D spheroid culture increased their pluripotency and altered the epigenetic status of pluripotency genes in MSCs (32). 3D spheroid culture of MSC significantly down-regulated the expression of SUV39H1. This resulted in reduced occupancy of the Nanog promoter region H3K9me3 (33). The reduction in H3K9me3 levels may be related to CYTOD. CYTOD reduces mRNA and protein levels of the EZH2, and reduced EZH2 expression reduces cellular H3K27me3 labeling (34).

In addition to extracellular environmental signaling due to 3D spheroid culture, extracellular mechanical stress signals can be translated into biochemical signaling events in the MSCs’ nucleus, thereby regulating MSCs activity. For example, MSCs cultured on elastic membranes with microgrooves have an elongated nuclear shape, reduced HDAC activity and increased histone acetylation compared to MSCs on flat substrates. Compression or stretch perpendicular to the microgroove resulted in reduced HDAC activity accompanied by increased histone acetylation and slight changes in nuclear shape during different types of mechanical force loading, suggesting anisotropic mechanical sensing in MSCs. Knockdown of nuclear matrix protein laminin A/C abolished the mechanical strain-induced changes in HDAC activity. Thus, micropatterning and mechanical strain on the substrate can modulate nuclear shape, HDAC activity and histone acetylation in an anisotropic manner, and the nuclear matrix mediates the mechanical transition (35). Cyclic mechanical stretch (CMS) promotes osteoblastogenesis in MSCs *in vivo* and *in vitro*. DNMT3B binds to the Shh gene promoter, leading to mechanical unloading of DNA hypermethylation in MSCs. Mechanical stimulation down-regulates the protein levels of DNMT3B, leading to DNA demethylation and SHH expression. Mechanical stimulation regulates osteoblast gene expression by directly regulating DNMT3B (36). Indeed, epigenetic modifications are involved in storing these mechanical cues, regulating gene expression, and ultimately leading to mechanical memory. Using hydrogels containing allyl sulfur crosslinkers and free radical-mediated addition-break chain transfer processes to achieve *in situ* softening of MSC-loaded hydrogels at different time points, the results show that histone acetylation and chromatin organisation adapt rapidly after softening, which can be reversible or irreversible depending on the time of exposure to the hard microenvironment. Epigenetic remodeling may be durable and may be a memory retainer (37).

### 3.2 Epigenetic regulation of MSCs *in vivo*

Different DNA methylation modification patterns of MSCs from different tissue origins may be responsible for their various osteogenic abilities (38). Dental pulp stem cells (DPSCs), periodontal ligament stem cells (PDLSCs), and dental follicle progenitor cells (DFPCs) have different osteogenic potentials. They differ in the methylation levels of specific genes associated with bone formation (genes for biological processes associated with osteogenesis, such as collagen fibril organization, trabecular formation, and calcium ion processes, as well as DNA encoding cell surface antigens, microRNAs, and transcription factors in osteogenesis-related pathways) (39). During the osteogenic differentiation of MSCs, the expression level of DNMT and TET changes in response, which lead to DNA methylation fluctuation (40). The average genomic methylation levels and CpG methylation levels in transcription factor regions (TFs) were increased, with CpG methylation levels in various genomic elements mainly in the middle-high methylation section, and CpG methylation levels in the repeat element had highly methylated levels (41). Disordered DNA methylation regulation can impair the osteogenic differentiation capacity of MSCs. Notably, though rare, the deregulation may be tissue-specific. It may represent up- or down-regulation in MSCs from different tissue origins.

Specifically, DNMT can negatively regulate MSC-mediated tissue regeneration, as DNMT can be consistent with osteogenesis or angiogenesis after allografting (42). TET is also closely associated with MSC homeostasis, and several knockout models suggest a role for TET in the maintenance of bone homeostasis (10). The epigenetic state of MSCs is associated with the biased differentiation plasticity towards its tissue of origin, proposing a mechanism related to the retention of epigenetic memory. For example, in one research reported by Ng et al., down-regulation of DNA methylation levels can cause high osteogenic differentiation capacity in MSCs derived from bone tissue and impaired osteogenic differentiation capacity in MSCs derived from adipose tissue (43). This osteogenic capacity impairment of MSCs disturbed by DNA methylation is one of the pathogenesis of several diseases with abnormal bone homeostasis, including steroid-related osteonecrosis of the femoral head (ONFH), osteogenic impairment due to pediatric aplastic anemia (AA), senile osteoporosis, oxidative stress-induced osteoporosis, and diabetic osteoporosis (44–48). All above fully illustrates the critical role of DNA methylation in regulating the osteogenic differentiation function of MSCs.

Histone modifications are essential for bone development and homeostasis. Knockout of histone-modifying enzymes causes abnormalities in the development of the skeletal system (49), and histone-modification abnormalities are involved in the pathogenesis of abnormal bone metabolic diseases such as osteoporosis (50). Histone modifications are dynamically adjusted during osteogenesis toward differentiation in MSCs to provide dynamic and fine-tuned regulation of critical genes required for various processes of osteogenesis. During MSCs proliferation, early commitment, matrix deposition, and mineralization phases, histone modification has temporal changes; therefore, loss or gain of specific histone modifications is the primary predictor (51). Indeed, the regulation of MSCs osteogenic capacity by histone modifications is related to the transcriptional regulation of the promoters of genes involved in osteogenesis, including RUNX2, SP7, FOXO1, etc. Histone modifications can also regulate osteogenesis by regulating key pathways such as the Wnt/β-catenin signaling pathway (52, 53). There are various types of histone modifications, and later, we mainly introduce the effects of histone methylation modification and acetylation modification on MSCs osteogenic differentiation.

### 3.3 Effect of DNA methylation of MSCs in bone regeneration

#### 3.3.1 DNA methylation

In the human genome, about 1% of the cytosine bases in DNA are modified by methylation, which often occurs in the CpG island-rich regions of genes with regulatory sequences (38). Although other types of modifications, such as N6-deoxyadenosine methylation (6mA), have been reported (39), the best-characterized type of DNA modification is 5-methylcytosine (5mC) (40). This modification process is reversible, with DNA methyltransferase (DNMT) catalyzing the covalent binding of methyl to cytosine and the ten-eleven translocation (TET) enzyme family catalyzing DNA hydroxymethylation and its subsequent demethylation by oxidizing 5mC to 5-hydroxymethylcytosine (5hmC). This dynamically balanced epigenetic regulation has a vital role in the regulation of gene expression in cells because DNA methylation can interfere with the recognition sites of transcription factors and thus regulate gene transcription, or it can transregulate gene expression through the action of methyl-CpG-binding domain proteins (MBD). DNA methylation is generally considered a modification process that promotes gene silencing. CpG sequences of genes that are not expressed or expressed at low levels are usually hypermethylated, while the opposite is true for actively expressed genes.

The normal regulation of DNA methylation is closely associated with the development and maintenance of homeostasis. Its regulation is involved in developing the nervous system, the retina, etc. (41, 42). Given its close relationship with cellular senescence, clocks mapped by DNA methylation can be an estimator of aging (43). However, dysfunctional DNA methylation leads to immunodeficiency, centromeric instability, facial abnormality (ICF), and tumorigenesis (44–46). These pathogenic effects indicate the role of DNA methylation in basic cellular life activities such as cell cycle regulation, fate determination, cellular senescence, etc. (47–49).

#### 3.3.2 DNA methylation on MSCs differentiation in bone regeneration

##### 3.3.2.1 Osteogenic differentiation

Different DNA methylation modification patterns of MSCs from different tissue origins may be responsible for their various osteogenic abilities (50). For instance, dental pulp stem cells (DPSCs), periodontal ligament stem cells (PDLSCs), and dental follicle progenitor cells (DFPCs) have different osteogenic potentials. They differ in the methylation levels of specific genes associated with bone formation. These genes for biological processes are associated with osteogenesis, such as collagen fibril organization, trabecular formation, and calcium ion processes, as well as DNA encoding cell surface antigens, microRNAs, and transcription factors in osteogenesis-related pathways (51). The response of DNMT and TET expression levels changes during the osteogenic differentiation of MSCs, causing fluctuations in DNA methylation (52). The average genomic methylation levels and CpG methylation levels in transcription factor regions (TFs) increase, with CpG methylation levels in various genomic elements mainly in the middle-high methylation section and CpG methylation levels in the repeat element showing highly methylated levels (53). However, disordered DNA methylation regulation can impair the osteogenic differentiation capacity of MSCs. Notably, although rare, the deregulation may be tissue-specific and may represent up- or down-regulation in MSCs from different tissue origins. The epigenetic state of MSCs is associated with the biased differentiation plasticity towards its tissue of origin, implying a mechanism related to epigenetic memory retention. For example, in one study by Ng et al., downregulation of DNA methylation levels can cause high osteogenic differentiation capacity in MSCs derived from bone tissues as well as impaired osteogenic differentiation capacity in MSCs derived from adipose tissues (54). When disturbed by DNA methylation, this impairment of osteogenic MSCs’ capacity is one of the pathogenesis of several diseases with abnormal bone homeostasis, including steroid-related osteonecrosis of the femoral head (ONFH), osteogenic impairment due to pediatric aplastic anemia (AA), senile osteoporosis, oxidative stress-induced osteoporosis, and diabetic osteoporosis (55–59). All these illustrate the critical role of DNA methylation in regulating the osteogenic differentiation function of MSCs.

The methylation of promoters or enhancers of osteogenesis-related genes orchestrates the osteogenesis of MSCs, while the DNMT family mediates DNA methylation. For instance, DNMT1 mediates the maintenance of DNA methylation, which occurs when one strand of the double-stranded DNA is already methylated and the other is not. DNMT1 can affect osteogenesis by regulating the expression of various osteogenesis-related genes. The reduced expression of DNMT1 prevents aberrant DNA methylation of NOTCH1 and NOTCH2, leading to their transcriptional upregulation and thus promoting enhanced osteogenic differentiation of MSCs (60). Moreover, DNMT1 mediates HOXA2 regulation through HOTAIRM1, which represses overall DNMT1 expression, leading to hypomethylation and HOXA2 induction, thereby promoting osteogenesis in MSCs (61). Furthermore, DNMT1 mediates hypermethylation of the MEG3 promoter in MSCs, decreasing the expression of MEG3 and inhibiting the transcriptional activity of BMP4, ultimately suppressing osteogenic differentiation (56). Unlike DNMT1, DNMT3A and DNMT3B catalyze *de novo* methylation, which occurs when neither double-stranded DNA is methylated. Furthermore, this type of DNA methylation regulates the osteogenic function of MSCs by affecting the expression of osteogenic-related genes. In addition, the overexpression of DNMT3A eliminates H_2_O_2_ treatment-induced hypomethylation of ALP and RUNX2 promoter regions, while the DNMT inhibitor 5′-AZA-2′-deoxycytidine (5-AZA) reduces DNA methylation of ALP and RUNX2 in MSCs under oxidative stress as well as increases their expression, thereby enhancing osteogenic differentiation (62). In oxidative stress-induced osteoporosis, DNMT3B upregulates and triggers hypermethylation of KLF5 and reduces KLF5 expression, thus affecting *β*-catenin expression and its nuclear translocation, ultimately impairing osteogenesis in MSCs (58). Thus, the DNA methylation-promoting effect of DNMT is generally shown to be an inhibitor of osteogenesis.

**Figure 2:**
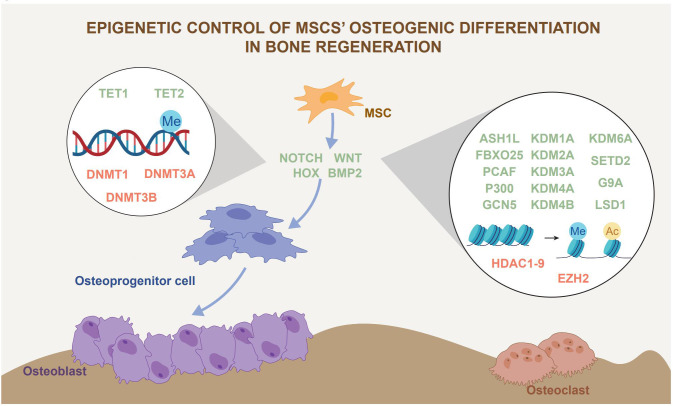
Epigenetic control of MSCs osteogenic differentiation in bone regeneration. Epigenetic regulation, represented by histone modification and DNA methylation, has an impact on MSCs osteogenic differentiation. Epigenetic regulation affects bone regeneration by influencing the lineage commitment of MSCs. The green font represents promoters of osteogenic differentiation and the red font represents inhibitors of osteogenic differentiation.

By oxidizing 5mC to 5hmC, TET catalyzes the hydroxymethylation and subsequent demethylation of DNA. *Tet1* ^-/-^; *Prx1* ^cre^ *Tet2* ^fl/fl^ showed significant bone loss and a double knockout (*Tet* DKO) of mice, but not in *Tet1* ^-/-^ mice. Although *Prx1* ^cre^ *Tet2* ^fl/fl^ mice also showed some bone loss, it was not as significant as in *Tet* DKO mice. BMSCs from *Prx1* ^cre^ *Tet2* ^fl/fl^ mice but not *Tet1* ^-/-^ mice have low osteogenic differentiation capacity. However, when compared to other groups, BMSCs from *Tet* DKO mice showed the lowest osteogenic capacity (10). This suggests a functional synergy between TET1 and TET2, with TET2 being more critical for the osteogenic function of BMSCs. However, in a study on the role of TET1/2/3 in MSCs osteogenesis using siRNA knockdown and retroviral-mediated forced expression of TET, osteogenesis is inhibited by TET1. Moreover, TET1 recruits the co-repressor protein SIN3A and the histone lysine methyltransferase EZH2 to osteogenic genes. On the other hand, although TET1 plays a role in this process, TET2 promotes osteogenesis and is directly responsible for the 5hmC levels of osteogenesis-related genes. Moreover, TET3 shows no functional role in differentiating bone formation in BMSCs (52). Furthermore, another independent study validated the osteogenesis-promoting effect of TET2 expression by *Tet2* ^-/-^mice and siRNA inhibition in MSCs *in vitro* (63). Although more evidence supports the osteogenic effect of TET2, the role of other subtypes of TET in osteogenesis needs further investigation. The roles of DNA methylation and histone modification in MSCs osteogenic differentiation ability in bone regeneration are summarized in **Figure 2**.

##### 3.3.2.2 Chondrogenic differentiation

Because cartilage scabs serve as scaffolds for mature bone regeneration during secondary bone healing, the osteogenic differentiation ability of MSCs is a critical factor in bone regeneration. Some recent studies investigated how DNA methylation affected the chondrogenic differentiation of MSCs, but they came to different conclusions.

In a mouse tibial fracture model, DNMT3B was expressed early in fracture healing, peaked ten days after fracture, and decreased to almost undetectable levels 28 days after fracture. The expression of DNMT3B is not only stage-specific but also cell-specific. For instance, high levels of DNMT3B are observed in chondrogenic lineage cells within the bone scab (64). Significant upregulation of DNMT3A and DNMT3B is consistently detected during chondrogenic differentiation of MSCs *in vitro* (65, 66). Moreover, DNMT3A overexpression increases gene expression of type II collagen more than 200-fold and significantly enhances chondrogenesis in MSCs. In contrast, 5-AZA inhibits the chondrogenic differentiation of MSCs. At the same time, loss-of-function assays with siRNAs targeting DNMT3A significantly inhibit chondrogenic differentiation in MSCs. Thus, DNA methylation has a crucial role in regulating chondrogenic differentiation in MSCs (65).

However, in another study, the chondrogenic differentiation of MSCs exhibited DNA hypomethylation at many key cartilage gene loci (Barter et al., 2020). The R-enantiomer of 2-hydroxylglutarate (R-2HG) inhibits the chondrogenic differentiation of MSCs. On the other hand, R-2HG induces a significant DNA hypermethylation state in MSCs. By inducing DNA hypermethylation, R-2HG may dysregulate the chondrogenic differentiation of MSCs (67).

On the other hand, during chondrogenic differentiation of MSCs, TET1 was highly expressed but gradually decreased. *In situ* hybridization using specific RNA probes on frozen sections of 15-day-old mouse embryos shows a strong signal in the chondrogenic embryonic skeleton. When 5-AZA is applied in the early stages of cartilage formation, cartilage-specific gene expression and cartilage formation are reduced. In contrast, when 5-AZA is added to differentiated chondrocytes, there is a stimulatory effect, and the DNA methylation patterns of essential chondrogenic marker genes are altered. In other words, TET1-induced DNA demethylation plays a vital role in cartilage formation, while inhibition of DNA methylation plays different roles in different stages of cartilage formation *in vitro* (68).

#### 3.3.3 Effects of DNA methylation on MSCs proliferation

DNMT showed a proliferation-promoting effect on MSCs. Knocking down the DNMT1 level in MSCs reduced the cell proliferation rate. At the same time, overexpression of DNMT1 increased the proliferation rate of MSCs (69). On the other hand, TET had an inhibitory effect on cell proliferation. *Tet* DKO and *Prx1* ^cre^ *Tet2* ^fl/fl^ BMSCs increased the proliferation rate. Knockdown of *Tet1* and *Tet2* expression using siRNA also induced a higher proliferation rate (10, 70). The positive effect of DNA methylation on the proliferation of MSCs is closely related to their regulation of the cell cycle. CDKN1A is a member of the family of cell cycle protein-dependent kinase inhibitors that regulate cell cycles and coordinate DNA replication and repair by inhibiting the complex activity of cyclin-dependent kinases (CDKs). CDKN1A is critical for regulating the G1/S transition and can be regulated by DNA methylation. In a study by Lu et al., OCT4 promoted proliferation, cell cycle progression, and the osteogenic differentiation capacity of MSCs. MSCs with overexpressed OCT4 have a shortened G1 phase and extended G2 and S phases, which are associated with the elevated DNMT gene expression by OCT4. Furthermore, the administration of 5-AZA and zebuline resulted in a dose-dependent and time-course-related inhibition of DNMT activity. At the same time, CDKN1A was significantly elevated. In addition, the proliferation-related proteins CCND1, PCNA, and Ki67 were downregulated. In other words, OCT4 maintains the self-renewal ability of MSCs by upregulating DNMTs to suppress CDKN1A expression (49).

#### 3.3.4 Effects of DNA methylation on MSCs aging

The term “cell senescence” refers to cellular proliferation, differentiation capacity, and physiological functions that gradually decline over time. Increasing evidence shows that MSCs senescence perpetuates aging or age-related diseases (27). In osteoporosis due to aging, MSCs have been reported to have a critical role in regulating bone mass (71). Senescence can also significantly affect the clinical applications of MSCs. Since cell senescence is consistent with body aging, senescent MSCs impair multipotent abilities, immune regulation, and cell proliferation abilities (72, 73). In addition, the therapeutic function of MSCs can be impaired in the transplants of autologous MSCs in elderly patients. On the other hand, since *in vitro* expansion is unavoidable before the clinical use of MSCs, prolonged cell expansion may lead to MSCs senescence. As a result, regulation of MSCs senescence may be critical in treating aging-related diseases and ensuring high-quality applications of MSCs.

Fernández et al. collected MSCs from individuals aged 2 to 92 years and analyzed their DNA methylation, finally identifying 18,735 hypermethylated and 45,407 hypomethylated CpG sites associated with aging. As in differentiated cells, hypermethylated sequences were enriched for chromatin repression marks, while hypomethylated CpG sites were strongly enriched in the active chromatin mark H3K4me1. This suggests a cell type-independent chromatin signature of DNA methylation during aging. Moreover, their findings indicate that the dynamics of DNA methylation during the aging process depend on a complex mix of factors that include the DNA sequence, cell type, and chromatin context involved and that, depending on the locus, the changes can be modulated by genetic and/or external factors (74). Furthermore, a study of age-related changes in 5hmC identified 785 high and 846 hypo-hydroxymethylated CpG sites in MSCs obtained from older individuals. DNA hyper-hydroxymethylation in the senior group was associated with a loss of 5mC, suggesting that at specific CpG sites, this epigenetic modification may play a role in DNA methylation alteration over time (75).

Some studies focused on how DNA methylation regulates the senescence of MSCs. For instance, Yang et al. analyzed the effects of aging on MSCs using three types of dental pulp-derived MSCs (DPSCs), including stem cells from exfoliated deciduous teeth (SHEDs) and permanent teeth of young (Y-DPSCs) and old (A-DPSCs) humans. The study found that the stem cell differentiation capacity of DPSCs decreased with age. Moreover, expression of the serine metabolism-related enzymes phosphoserine aminotransferase 1 (PSAT1) and phosphoglycerate (PHGDH) is reduced in A-DPSC. This provides less methyl donor S-adenosylmethionine (SAM) for DNA methylation, leading to hypomethylation of the senescence marker CDKN2A and resulting in DPSC senescence (76). Extended *in vitro* expansion significantly affected the DNA methylation distribution of MSCs. Compared to primary cells, 14 days of culture expansion led to 4831 significantly differentially methylated sites. These differences were located in genes involved in plasma membrane composition, cell adhesion, and transmembrane signaling (77). The precise detection of MSCs senescence in *in vitro* culture is of great interest for the clinical application of MSCs. DNA methylation can be applied as a quality control tool in large-scale industrial production. Because DNA methylation changes triggered by the prolonged expansion of MSCs are stable and reproducible, CpG islands that become continuously hypermethylated or hypomethylated during long-term MSCs’ culture have been identified. Furthermore, changes in DNA methylation during expansion are not directly regulated by the target mechanism but rather resemble epigenetic drift (78).

Several studies reported on the use of drugs to modulate the senescence of MSCs and thus improve their stemness and differentiation abilities. For instance, DNMT inhibitor RG108-treated MSCs increased expression of the anti-senescence genes TERT, VEGF, and ANG and reduced expression of the senescence-related genes ATM and CDKN1A. Furthermore, RG108-treated MSCs reduced SA-*β*-galactosidase activity and senescence protein expression (79–81). Another DNMT inhibitor, 5-AZA, has been reported to inhibit MSCs senescence by reducing DCN mRNA expression and CGI-I methylation, which ultimately enhances the proliferation of serum-starved, aged BMSCs under IGF-I stimulation (82).

#### 3.3.5 DNA methylation crosstalk in MSC functions

Epigenetic regulation of MSCs is complex; different types of epigenetic regulation have crosstalk and can exert their regulatory effects on MSCs. For instance, DNA methylation and histone modifications have crosstalk. KDM4A expression and adipogenesis in MSCs are upregulated during osteogenesis. Moreover, the overexpression of wild-type KDM4A blocks the osteogenic differentiation of progenitor cells. In contrast, the absence or inactivation of KDM4A in undifferentiated progenitor cells promotes osteoblast differentiation. Furthermore, KDM4A overexpression upregulated the expressions of the secretory frizzled-related protein (SFRP4) and of the CCAAT/enhancing binding protein alpha (C/EBPα). Chromatin immunoprecipitation analysis showed that KDM4A directly binds to SFRP4 and C/EBPα promoters, removes the histone methylation marker H3K9me3, and reduces the DNA methylation levels of CpG in the promoter regions of C/EBPβ and SFRP4 (83).

DNA methylation and non-coding RNAs also have crosstalk. For example, LncRNA-AK137033 expression, DNA methylation levels in the sFrp2 promoter region, Wnt signaling pathway markers, and osteogenic differentiation potential are all reduced in MSCs isolated from diabetic osteoporosis (DOP) bone-deficient mice. In contrast, silencing AK137033 inhibited the Wnt signaling pathway and osteogenic capacity of CON MSCs by reducing the DNA methylation level of the sFrp2 promoter region (84).

Even these three epigenetic modifications have crosstalk. For example, in hind limb unloading (HLU) rats, H3K9 methylation and its cross-talk with DNA methylation have a key role in regulating lncH19 expression and bone loss in this disease model. Expression of G9a, ubiquitin-like PHD, the structural domain of the ring finger 1 (UHRF1), and DNA methylation transferase 1 (DNMT1) increased in HLU rats, together with increased levels of lncH19 promoter histone H3 lysine 9 (H3K9) d/trimethylation. Alterations in G9a, DNMT1, or UHRF1 expression significantly affected lncH19 levels and osteogenic activity in UMR106 cells. Moreover, osteogenic gene expression and matrix mineralization were strongly promoted after the simultaneous knockdown of G9a, DNMT1, and UHRF1. The lncH19 promoter has physical interactions with G9a and DNMT1 and direct interactions with DNMT1, G9a, and UHRF2. The overexpression of DNMT1, G9a, or UHRF1 enriched H3K9me2/me3 and 5-methylcytosine at the lncH19 promoter, respectively. *In vivo* rescue experiments showed that the knockdown of DNMT1, G9a, or UHRF1 significantly attenuated bone loss in HLU rats (85).

### 3.4 Effects of histone modifications of MSCs on bone regeneration

#### 3.4.1 Histone modifications

In eukaryotic cells, chromatin is a highly ordered, dense structure that restricts the contact and binding of transcription factors to DNA and controls gene transcription. Gene transcription begins by unraveling the dense structure of chromatin and opening the DNA around histones to facilitate the binding of transcription factors, thereby facilitating gene transcription. Covalent chemical modification of histones is a form of epigenetic regulation of gene expression by altering the spatial structure of chromatin. They usually occur on core histones H3 and H4 of nucleosomes, including acetyl, methyl, phosphate, ubiquitin, and poly-ADP ribosyl groups, which various motifs can modify. For example, histone acetylation is one of the most common forms of modification that occurs at H3 and H4 lysine residues. In the presence of histone acetyltransferase (HAT), an acetyl group is added to the lysine at the N-terminal end of the histone. At this point, the histone affinity for DNA is reduced, making DNA more favorable for the entry of transcription factors and thus promoting gene expression. This process can be reversed by histone deacetylase (HDAC). Histone modifications regulate gene expression and affect several biological processes, including DNA repair, cell cycle, stemness, changes in cell states, genome stability, and nuclear architecture (86, 87). In pathological states, histone modification remodeling is a prominent feature of cancer (88) and the pathogenic progression of autoimmune diseases, cardiovascular aging and diseases, neurodegenerative disease, etc. (89, 90).

#### 3.4.2 Effects of histone modifications on osteogenic MSCs differentiation

Histone modifications are essential for bone development and homeostasis. The knockout of histone-modifying enzymes causes abnormalities in the development of the skeletal system (91). Moreover, histone-modification abnormalities are involved in the pathogenesis of abnormal bone metabolic diseases such as osteoporosis (92).

During osteogenesis, histone modifications are dynamically adjusted toward differentiation in MSCs to provide dynamic and fine-tuned regulation of critical genes required for various processes of osteogenesis. Histone modification undergoes temporal changes during MSCs proliferation, early commitment, matrix deposition, and mineralization. Therefore, the loss or gain of specific histone modifications is a primary predictor (93). Indeed, the regulation of MSCs’ osteogenic capacity by histone modifications is related to the transcriptional regulation of the promoters of genes involved in osteogenesis, including RUNX2, SP7, FOXO1, etc. The various types of histone modifications can regulate osteogenesis by regulating key pathways such as the Wnt/β-catenin signaling pathway (94, 95). This paper introduces the effects of histone methylation modification and acetylation modification on osteogenic MSCs differentiation. The roles of histone modification in MSCs' osteogenic differentiation are summarized in **Table 1**.

**Table 1:** Histone modification in MSCs’ osteogenic differentiation.

Enzyme	Histone modification reported	Type of MSCs (Species)	Mechanism	Ref.
SETD2	H3K36me3	BMSCs (Mouse)	H3K36me3 mediated by SETD2 regulates the cell fate of mesenchymal stem cells (MSCs) *in vitro* and *in vivo*. Deficiency of SETD2 in BMSCs resulted in bone loss and bone marrow obesity.	(96)
	H3K36me3	PDLSCs (Human)	The splicing factor heterogeneous nuclear ribonucleoprotein LhnRNPL inhibits osteogenic differentiation of PDLSCs through downregulation of the H3K36me3-specific methyltransferase SETD2.	(97)
FBXO25	H3K4me3	UCMSCs (Human)	FBXO25 increased monoubiquitination of H2BK120 and subsequently promoted H3K4me3 thereby inducing transcription of the key transcription factor OSX and increasing the expression of downstream osteoblast markers, OCN, OPN, and ALP.	(98)
ASH1L	H3K4me3	C3H10T1/2(Mouse)	ASH1L can regulate osteogenic differentiation by modifying the essential osteogenic transcription factor promoter region H3K4me3.	(99)
JMJD2B/KDM4B	H3K9me2	BMSCs (Human)	JMJD2B/KDM4B may induce osteogenic differentiation of BMSCs by regulating the methylation level of RUNX2 promoter H3K9me2.	(100)
G9A	H3K9me2	C3H10T1/2 (Mouse)	*Sox9* ^Cre^/*G9a* ^fl/fl^ mice show severe hypomineralization of the cranial vault bone, and G9A regulates cranial osteoblast proliferation and differentiation by binding to and activating RUNX2.	(91)
LSD1	Not given	ASCs (Human)	miR-137 negatively regulates osteogenic differentiation of ASCs through the LSD1/BMP2/SMAD4 signaling network.	(101)
	H3K4me2	(Mouse)	The absence of LSD1 in PRX1 lineage cells severely impairs fracture healing. LSD1 tightly controls retinoic acid signaling by regulating the level of AlDHA2 expression, which in turn regulates SOX9 expression.	(102)
KDM1A.	H3K9me3	BMSCs (Human)	KDM1A induces circ_AFF4, which promotes osteogenesis *via* IGF2BP3.	(103)
	Not given	SCAPs (Human)	Knockdown of KDM1A reduced ALP activity and mineralization, promoted the expression of bone differentiation markers and key transcription factors in SCAPs, and enhanced bone formation *in vivo*.	(104)
	Not given	SCAPs (Human)	HOXC8 negatively regulates the bone differentiation and migration capacity of SCAPs by directly enhancing KDM1A transcription.	(105)
KDM2A	H3K4me3 H3K36me2	SCAPs (Human)	Inflammatory or hypoxic conditions promote the expression of KDM2A and inhibit SFRP2 transcription by reducing histone methylation in the SFRP2 promoter, which in turn promotes the typical Wnt/β-catenin signaling pathway.	(106)
KDM3A	H3K9me1 H3K9me2 H3K9me3	BMSCs (Rat)	KDM3A enhances ERK2 and KLF2 expression by promoting their demethylation, which further promotes osteogenic differentiation.	(107)
KDM4A	H3K9me3	BMSCs (Mouse)	KDM4A directly bound the promoters of SFRP4 and C/EBPα, removed the histone methylation marker H3K9me3, and reduced DNA methylation in the promoter regions of C/EBPα and CpG of SFRP4 levels.	(83)
KDM4B	H3K9me3	BMSCs (Mouse)	KDM4B deficiency in MSCs exacerbates skeletal aging and osteoporosis by increasing H3K9me3 to reduce bone formation and increase marrow adiposity.	(108)
		DMSCs (Human)	Direct and epigenetic activation of DLX5 by KDM4B through demethylation of H3K9me3.	(109)
KDM6A	H3K27me3	PDLSC (Human)	miR-153-3p inhibits PDLSC osteogenesis by targeting KDM6A and repressing ALP, RUNX2 and OPN transcription.	(110)
EZH2	H3K27me3	BMSC (Mouse)	The deletion of EZH2 in BMSCs inhibits osteogenic differentiation and impedes cell cycle progression, as evidenced by reduced metabolic activity, decreased cell numbers, changes in cell cycle distribution, and expression of cell cycle markers.	(111)
P300	H3ac	C3H10/C2C12 (Human)	P300 promotes the acetylation of histone 3 and the formation of a transcriptional complex with RUNX2 to enhance osteogenesis.	(112)
	H3K27ac	MenSCs UCMSC (Human)	P300 is recruited to the RUNX2 promoter with the help of c-Jun, which promotes H3K27ac at this site and ultimately promotes osteogenesis	(113)
	H3K9ac H3K27ac	BMSCs (Rat)	Ethanol promotes early growth response factor 1 (Egr1) expression and nuclear translocation by recruiting P300 and binding directly to the ACE promoter region, and Egr1 is involved in promoting histone acetylation of ACE and subsequent RAS activation.	(114)
GCN5	H3K9ac	BMSCs (Mouse)	GCN5 promotes osteogenic differentiation of BMSCs by increasing H3K9ac on the Wnt gene promoter.	(115)
	H3K9ac H3K14ac	PDLSCs (Human)	GCN5 regulates the Wnt/β-linked protein pathway of PDLSCs by H3K9ac and H3K14ac in its promoter region to regulate DKK1 expression.	(116)
PCAF	H3K9ac	ADSCs BMMSCs (Human)	PCAF regulates BMP signaling by increasing H3K9ac.	(117)
HDAC1	Not given	BMSCs (Mouse)	The HDAC1-Wnt/β-Catenin signaling axis is involved in the antidiabetic bone loss effects of exendin-4, and HDAC1 may be a target of exendin-4.	(118)
HDAC1&2	H3ac	BMSCs (Human)	The inhibitory effects of HDAC’s H3ac-ability on BMSCs’ osteogenesis by HPL cells and HGFs are greater than those mediated by HDAC activity.	(119)
	H3K9ac	BMSCs (Human)	Dual small molecule inhibitors of HDAC1 and HDAC2 significantly increased H3K9ac in the promoter regions of OSX and ALP in MSC, thereby enhancing MSCs’ osteogenesis.	(120)
HDAC1 SIRT1&2	Not given	ADSCs (Human)	Melatonin and vitamin D can regulate the commitment of ADSCs to the osteogenic phenotype by upregulating HDAC1, SIRT1, and 2.	(121)
HDAC2	H3K27ac	UCMSCs (Human)	HOXA-AS2 knockdown leads to transcriptional repression of the master osteogenic transcription factor SP7 through an NF-κB/HDAC2 coordinated H3K27 deacetylation mechanism.	(122)
HDAC2&3	Not given	BMSCs (Human)	Selective inhibitors of HDAC2&3 promote osteogenic differentiation of BMSCs *in vitro* and *in vivo*.	(123)
	H3K9ac	DPSCs (Human)	HDAC2&3 selective inhibitor pretreatment significantly upregulated the expression of osteoblast-related genes and proteins in DPSCs during osteogenic differentiation.	(124)
HDAC3	Not given	BMSCs (Human)	miR-4286 may act through a tight osteogenic angiogenic pathway to attenuate alcohol-induced bone loss by targeting HDAC3.	(125)
HDAC4	Not given	HMSCs BMSCs (Human)	miRNA-19a-3p promoted osteogenic differentiation of MSCs by suppressing HDAC4 expression, thereby alleviating the progression of osteoporosis.	(126)
	H3K27ac	BMSCs (Rat)	Upregulation of HDAC4 expression in BMSCs can inhibit their osteogenic differentiation by suppressing H3K27 acetylation in the IGF1 promoter region.	(127)
HDAC5	Not given	BMSCs (Mouse)	miR-2861 suppresses HDAC5 expression and promotes osteogenic differentiation.	(128)
HDAC6	H3K9/K14ac H4K12 ac	BMSCs (Mouse)	Accumulation of HDAC6 and hypoacetylation of histones on the RUNX2 promoter contribute to the diminished osteogenic differentiation potential of aging mouse bone marrow mesenchymal stem cells *in vitro*.	(129)
HDAC7	Not given	BMSCs HUVEC (Human)	miR-1260a enhances osteogenesis by inhibiting HDAC7.	(130)
HDAC8	H3K9ac	BMSCs (Human)	HDAC8 is associated with FD. HDAC8 is directly activated by CREB1 in FD BMSCs, and the cAMP-CREB1-HDAC8 pathway mediates FD pathogenesis.	(131)
HDAC9	Not given	PDLSCs (Human)	HDAC9 impairs the osteogenic differentiation ability of PDLSCs under inflammatory conditions.	(132)
	Not given	BMSCs (Human)	HDAC9 knockdown significantly inhibited osteoblast-specific gene expression and mineral deposition *in vitro*. The reduction in osteogenesis due to HDAC9 knockdown was partially rescued by MAPK signaling pathway activators.	(133)
	H3K9ac	BMSCs (Mouse)	HDAC9 regulates autophagy in BMMSCs by controlling H3K9 acetylation in the autophagy genes ATG7, BECN1, and LC3a/b promoters, which subsequently affects their genealogical differentiation.	(134)

Histone modifications in multiple MSCs that affect osteogenic differentiation of MSCs, their name, modification targets, tissue and species origin of MSCs, mechanism of action. Not given, not stated in original.

Abbreviations that do not appear in the main text. Egr, growth response factor; ACE, angiotensin-converting enzyme; RAS, renin-angiotensin systems; HPL, human periodontal ligament; HGFs, human gingival fibroblasts; FD, fibrous dysplasia; SCAPs, stem cells from apical papilla; SFRP, secreted frizzled-related protein; ERK, extracellular signal-regulated kinase; KLF2, KLF transcription factor 2; C/EBPα, CCAAT enhancer binding protein alpha; DLX, distal-less homeobox.

##### 3.4.2.1 H3K4 methylation modification is a positive osteogenic factor

In osteogenic-differentiated C3H10T1/2 cells, Ash1l is a histone 3 lysine 4 (H3K4) trimethyltransferase that was significantly upregulated. Ash1l affects the expression of essential osteogenic and chondrogenic transcription factors by modifying the trimethylation enrichment of H3K4 (H3K4me3) in their promoter regions (99). H3K4me3 and H3K9 acetylation were significantly enhanced in the WNT3A and DVL3 promoter sites. This histone modification mediates the osteogenic effect of ferutinin on MSCs (135). In addition, H3K4 methylation is subject to other epigenetic crosstalk that regulates the osteogenic differentiation of MSCs. For example, the overexpression of LncRNA ODIR1 can inhibit osteogenic differentiation of MSCs *in vitro* and *in vivo.* ODIR1 interacts with F-box protein 25 (FBXO25), and the latter increases the mono-ubiquitination of H2BK120 (H2BK120ub), which promotes H3K4me3, thereby promoting the osteogenic capacity of MSCs (98).

##### 3.4.2.2 H3K9 methylation modification is a negative osteogenic factor

Decreased methylation levels of the RUNX2 promoter H3K9me2 can induce osteogenic differentiation of BMSCs (100). The overexpression of KDM4A can promote osteogenic differentiation in BMSCs, knock down KDM4A, reduce the promoter expression levels of RUNX2, OSX, and OCN, and increase the expression level of H3K27me3. Thus, KDM4A has a crucial role in osteogenic differentiation and regulates the expression of osteogenic genes *via* H3K9me3 (136). Extracellular signals (e.g., Vitamin C) regulate the osteogenic differentiation of MSCs through H3K9me3. In the *Gulo* ^-/-^ mice model, where mice can only consume Vitamin C through exogenous supplementation, five weeks of Vitamin C deprivation elevated H3K9me3 and H3K27me3 marks in bone tissue. Furthermore, the knockdown of H3K9me3 demethylases KDM4A and KDM4B significantly downregulated the osteogenic capacity of MSCs (137).

##### 3.4.2.3 H3K27 methylation modification is a positive osteogenic factor

EZH2 is a key component of the polycomb repressor complex 2 (PRC2) complex that catalyzes histone methylation. In addition, EZH2 catalyzes the mono-, di-, and trimethylation of lysine 27 of histone H3. The deletion of EZH2 in mesenchymal progenitor cells (*Prx1* ^Cre^) disrupts intramembranous osteogenesis, leading to skeletal deformities. However, this study found that EZH2 negatively regulated osteogenic differentiation in MSCs. Moreover, this developmental abnormality of the skeletal system may be related to the diminished MSC proliferation and promotion of osteogenic differentiation triggered by the deletion of EZH2 in MSCs (11). Thus, EZH2 is essential in the early stages of MSCs’ lineage commitment (138). Using the temporally-inducible conditional deletion of EZH2 in the cranial neural crest cells (CNCC) and head paraxial mesoderm (PM)-derived cranial mesenchyme between E8.5 and E9.5 reduced the CNCC-derived calvarial bone and showed an almost complete loss of the PM-derived calvarial bone due to stagnation of calvarial fate commitment (139). The inhibitory effects of H3K27 methylation on osteogenesis brought about by EZH2 were associated with the Wnt/β-catenin signaling pathway. β-catenin was linked to the EZH2 promoter. β-catenin knockdown reduced EZH2 protein levels at the osteogenic motif and decreased H3K27me3. The antidifferentiation effect of β-catenin proteins was lost when EZH2 was inhibited (140). Furthermore, BMP2 was associated with the osteogenic inhibitory effect of EZH2. EZH2 inhibition promotes the induction of BMP2-mediated osteogenic differentiation of MSCs. In a mouse model of cranial critical size defects, the combination of BMP2 and GSK126 (an EZH2 inhibitor) produced better bone healing than a single treatment with either compound (141, 142). At the same time, EZH2-mediated methylation of H3K27 can be induced by external stimuli such as LPS. In such cases, EZH2 knockdown can attenuate TLR4/MyD88/NF-κB signaling to promote MSCs osteogenesis (143).

##### 3.4.2.4 H3K36 methylation is a positive osteogenic factor

The histone methyltransferase SET structural domain 2 (SETD2) catalyzes H3 lysine 36 trimethylation (H3K36me3). The *Prx1* ^Cre^ conditional deletion of *Setd2* in mouse BMSCs leads to bone loss and marrow adiposity. A deficiency of SETD2 in BMSCs *in vitro* promoted the differentiation tendency of adipocytes but not osteoblasts. Moreover, overexpression of lipopolysaccharide-binding protein (LBP) partially rescues impaired osteogenesis and enhances adipogenesis caused by the lack of SETD2 in BMSCs. Thus, SETD2 regulates the transcriptional Lbp initiation and elongation by promoting the trimethylation level of H3K36, which ultimately regulates the cell fate of MSCs at the ex vivo level (96). hnRNPL can downregulate SETD2, while SrCl2 stimulation can significantly downregulate the hnRNPL splicing factor. Thus, SrCl2 is involved in PDLC osteogenesis through the upregulation of H3K36me3 levels (97).

##### 3.4.2.5 Histone acetylation modifications are a positive factor in MSCs osteogenesis

Acetyltransferases such as P300 and GCN5 catalyze the acetylation modifications of histones, which promote the osteogenic differentiation of MSCs. Furthermore, c-Jun can recruit acetyltransferase P300 to the RUNX2 promoter and promote the acetylation of H3K27, thereby epigenetically activating RUNX2 gene transcription (113). Similarly, cryptochrome circadian regulatory (CRY) proteins 2 can regulate P300 to promote osteogenic differentiation through CLOCK/BMAL1/P300 signaling (112). GCN5 is another histone acetyltransferase that promotes osteogenic MSCs differentiation by increasing H3K9 acetylation on the WNT gene promoter. Furthermore, by acetylating H3K9 and H3K14 in its promoter region, GCN5 can regulate osteogenic differentiation *via* the Wnt/β-catenin pathway *via* the regulation of DKK1 expression. Reduced GCN5 expression inhibits Wnt signaling, leading to osteogenic failures in BMSCs from OVX mice (115, 116). Moreover, the H3K9 acetyltransferase PCAF plays a crucial role in MSCs osteogenesis. PCAF knockdown significantly reduces bone formation *in vitro* and *in vivo*. Mechanistically, PCAF controls the BMP expression of signaling genes by increasing H3K9 acetylation (117).

In line with the findings of acetyl modification, HDAC-mediated histone deacetylation presents negative osteogenesis regulation. The HDAC family contains several members, but due to the lack of specific inhibitors, these are not well-defined in many studies. Despite this, whether or not the HDAC subtypes are distinguished, histone deacetylation modifications are generally shown to impair osteogenesis. Reduced histone acetylation levels mediated by HDACs can mediate the conversion of extracellular physical signals into biochemical signaling events in the nucleus, affecting MSCs osteogenesis. MSCs respond to matrix sclerosis and eventually lead to the commitment of osteogenic fate by increasing nuclear tension and increasing histone acetylation through the deactivation of HDACs. Disrupting nuclear mechanoreception by separating the nucleus from the cytoskeleton can upregulate HDACs and prevent osteogenesis (35, 144). Pharmacological inhibition of HDAC activity in MSCs significantly elevated osteogenesis-related gene expression and markers. This osteogenic function-promoting effect exists both *in vitro* and *in vivo* (123). Studies on several HDAC subtypes, including HDAC1, HDAC2, HDAC3, HDAC4, HDAC5, HDAC6, HDAC7, and HDAC8 (119, 145–149), have confirmed these findings. In addition, reduced histone acetylation modifications triggered by other deacetylases have been reported to inhibit osteogenesis, such as the H3K9 deacetylase SIRT6, which regulates osteogenic differentiation of MSCs through the SIRT6-TRPV1-CGRP signaling axis (150).

#### 3.4.3 Effect of histone modifications on MSCs proliferation

Histone methylation modifications are closely related to the proliferative capacity of MSCs. Decreased MSCs proliferation due to obesity is correlated with the protein expression of global H3K4me3 but not with those of H3K9me3 and H3K27me3 (151). The inhibition of lysine demethylase 2A (KDM2A) expression using short hairpin RNAs inhibited MSCs proliferation and arrested cell cycle progression at the G1/S phase in MSCs. KDM2A silencing increased H3K4me3 at the cyclin-dependent kinase inhibitor 2B (CDKN2B) and cyclin-dependent kinase inhibitor 1B (CDKN1B) motifs and regulated its expression, resulting in the regulation of cell proliferation (152). MSCs proliferation can also be consistently inhibited by the knockdown of SNRNP200, a co-binding factor of KDM2A, by blocking MSCs in the G2/M and S phases of the cell cycle. Deleting SNRNP200 upregulated CDKN1A and p53 but downregulated CDK1, CYCB, CYCE, and CDK2 (153). Pharmacological G9a inhibition significantly enhanced MSCs proliferation (154).

Tan et al. used ChIP-on-chip to generate genome-wide histone H3K9 acetylation and dimethylation profiles on gene promoters in human bone marrow MSCs. The functional analysis suggests that many critical cellular pathways in human bone marrow MSCs self-renewal (e.g., classical signaling pathways, cell cycle pathways, and cytokine-related pathways) may be regulated by H3K9 acetylation modifications. These data suggest that gene activation and silencing affected by H3K9 acetylation and demethylation may be essential to maintaining the self-renewal and multi-potency of human bone marrow MSCs (155). Pharmacological inhibition of HDACs increased the acetylation of histones H3 and H4, activated CDKN1A transcription, and ultimately blocked the cell cycle in the G2/M phase (156). The deletion of EZH2 in MSCs impedes cell cycle progression, as proven by changes in cell cycle distribution and expression of cell cycle markers. RNA Seq analysis of EZH2 cKO calvaria showed that the cell cycle protein-dependent kinase inhibitor CDKN2A was the most significant cell cycle target of EZH2, which plays a dual role in bone formation by inhibiting osteogenic lineage commitment while promoting the proliferation and expansion of osteoprogenitor cells (111). The HDAC inhibitor (HDACi) significantly improved the proliferation of MSCs and delayed the senescence of MSCs. HDACi can affect histone H3K9 or H3K14 acetylation and H3K4 dimethylation, thereby increasing the mRNA expression of pluripotency and proliferation genes and inhibiting the spontaneous differentiation of MSCs (157).

#### 3.4.4 Effects of histone modifications on MSCs aging

##### 3.4.4.1 Histone methylation in MSCs aging

H3K27me3 and H3K9me3 positive MSCs of bone marrow were significantly increased in senescent mice (158). During stem cell senescence, the level of cryptic transcripts is elevated; the increase in cryptic transcripts promotes stem cell senescence. In MSCs, the decrease in H3K36me3 and the increase in H3K4me1, H3K4me3, and H3K27ac are distinct chromatin features of regions with age-related cryptic transcripts (159, 160), suggesting that this histone modification may be a promoter of aging.

Specific studies on histone methylation in MSCs further illustrate the relationship between histone methylation modifications and stem cell senescence. For instance, H3K9 demethylated KDM3A, while KDM4C promoted heterochromatin reorganization, attenuated the DNA damage response, and delayed MSCs senescence. MSCs from *Kdm3a* ^-/-^ mice had chromosomal tissue defects, increased DNA damage response, and accelerated bone aging. Human bone marrow MSCs and transcriptome database analysis revealed a consistently negative association of KDM3A and KDM4C with aging (161). KDM4B deletion in MSCs exacerbated skeletal aging and osteoporosis, thus reducing the bone formation and increasing marrow adiposity by elevating H3K9me3. Furthermore, KDM4B ablation impaired MSCs self-renewal and promoted MSCs failure by inducing the formation of senescence-associated heterochromatin foci (108). The inhibitory effects of KDM4B on MSCs senescence in oral and maxillofacial MSCs (OMSCs) are also confirmed. KDM4B knockdown significantly impaired the OMSCs proliferation rate in the early and late passages. Furthermore, senescence-associated *β*-galactosidase (SA-*β*-gal) staining revealed that KDM4B ablation promoted senescence in OMSCs with elevated CDKN2A and CDKN1A gene expressions in late passages. KDM4B loss promotes adipogenesis and OMSCs aging, which further impairs the bone lipid balance in the mandible (162). EZH2 enhances the modification of H3K27me3 on the FOXO1 promoter and inhibits its function in activating downstream genes in antioxidant defense. Therefore, EZH2 overexpression reduces antioxidant enzyme levels and causes excessive oxidative damage in aged BMSCs, resulting in defective bone formation and regeneration (163). These findings clearly show that histone methylation modifications promote MSCs aging.

##### 3.4.4.2 Histone acetylation in MSCs aging

P300 can regulate the senescence of MSCs associated with activating the P53/CDKN1A signaling pathway. In late-passage senescent MSCs, P300 was significantly reduced, but P53 and CDKN1A levels were elevated. This is confirmed by P300 knockdown and inhibition of acetyltransferase (KAT) activity *via* C646. The inhibition of P300 induced senescence and reduced the proliferative potential of MSCs (164).

During the long-term culture of MSCs *in vitro*, HDAC activity and HDAC4, HDAC5, and HDAC6 expression gradually increased, while histone H3/H4 acetylation was down-regulated (147). HDAC6-mediated histone hypoacetylation in the *Runx2* promoter diminished osteogenesis in aging mouse BMSCs. HDAC6 inhibition mediated by siRNA or inhibitors activated RUNX2 expression, rescued the *in vitro* osteogenic potential of BMSCs, and attenuated age-related bone loss in mice *in vivo* (129). Mechanistically, the effects of HDAC6 on MSCs senescence were associated with a significant increase in CDKN1B protein levels when inhibiting HDAC6. Moreover, HDAC6 interacted with CDKN1B and deacetylated CDKN1B. CDKN1B acetylation is negatively regulated by HDAC6, which correlates with changes in CDKN1B protein levels (165).

## 4 Conclusions

MSCs closely regulate bone regeneration. Epigenetic regulation alters the osteogenesis capacity of MSCs in bone regeneration by influencing the osteogenic differentiation, proliferation, and senescence of MSCs. Although epigenetic regulation of MSCs orchestrates bone regeneration, more preclinical experiments are needed to evaluate the post-epigenetic regulation of MSCs.

## Author contributions

FY conceived, wrote, revised the manuscript, and made the figure and table. XW and FY revised the manuscript. LY and FY reviewed, revised, and edited the manuscript. All authors read and approved the final manuscript.
